# Small Airway Function as an Indicator for Persistent Airflow Limitation Asthma: A Retrospective Study

**DOI:** 10.1111/crj.70174

**Published:** 2026-03-07

**Authors:** Zhongzhao Wang, Xintong Du, Yang Luo, Heng Gong, Hao Tang

**Affiliations:** ^1^ Department of Respiratory and Critical Care Medicine, Changzheng Hospital, Second Affiliated Hospital of Naval Medical University Shanghai China; ^2^ Department of Respiratory and Critical Care Medicine, Changhai Hospital, First Affiliated Hospital of Naval Medical University Shanghai China; ^3^ School of Basic Medicine, Naval Medical University Shanghai China

**Keywords:** asthma, lung function examination, persistent airflow limitation, small airway function

## Abstract

**Background:**

Asthma is a heterogeneous disease characterized by chronic inflammatory changes in the airways and reversible airflow limitation. Persistent airflow limitation (PAL) asthma, as a common phenotype of asthma, is usually defined as forced expiratory volume in 1 s/forced vital capacity (FEV1/FVC) ratio lower than 0.7 after bronchodilator in asthmatic patients. Recent research has indicated a correlation between PAL asthma and more frequent asthma exacerbations.

**Methods:**

A total of 1322 asthma patients were retrospectively assessed, and finally, 441 asthma patients were included in the study. Among them, 217 patients were diagnosed with non‐PAL asthma, and 224 patients were PAL asthma. The differences in basic information, clinical manifestations, lung function, and laboratory test indicators between the PAL asthma and non‐PAL asthma groups were compared. A clinical prediction model for PAL asthma based on patient demographics was established using logistic regression analysis.

**Results:**

Comparing the demographic data of patients with PAL and non‐PAL asthma, it was observed that PAL asthma was more common in elderly smoking males. Patients with PAL asthma had a higher severity of asthma, poorer control, and lower life quality compared to non‐PAL asthma. In terms of lung function, patients with PAL asthma mainly exhibited obstructive ventilatory impairment, small airway dysfunction, diffusion dysfunction, and increased residual volume. Notably, after bronchodilators, patients with PAL asthma showed a significantly lower rate of improvement in small airway function compared with non‐PAL asthma. Logistic regression analysis showed gender, smoking index, and Asthma Control Test (ACT) score were independent risk factors for PAL asthma. Linear regression analysis showed small airway function was closely associated with ACT score.

**Conclusion:**

PAL asthma was poorly controlled, leading to a diminished quality of life. The limited improvement in small airway function after bronchodilator in PAL asthma suggested that targeting small airway dysfunction may hold significant value in the treatment of PAL asthma.

This retrospective study (441 asthma patients) found that PAL asthma is more common in elderly males, with smoking index and ACT score as its independent risk factors. PAL patients have poor small airway function and limited improvement after bronchodilators, suggesting targeting small airways may effective for PAL asthma treatment.

## 1 Introduction

Asthma is a heterogeneous disease characterized by chronic airway inflammation and reversible airway obstruction [1]. An epidemiological investigation conducted in China from 2012 to 2015, involving a cohort of over 50 000 adults, identified an adult asthma prevalence of 4.2%, with persistent airflow limitation (PAL) asthma constituting approximately 25% of asthma [2]. Furthermore, in another study, PAL asthma was present in up to 33% of asthma patients, across a spectrum from mild to severe cases [3]. Some scholars suggested that PAL asthma is linked to poor asthma control, reduced lung function, and frequent exacerbations [4]. However, the understanding of PAL asthma remains insufficient. Experts have highlighted the prevalent underdiagnosis and undertreatment of asthma in China [5]. Consequently, the early recognition and tailored management of PAL asthma are crucial, underscoring the urgent need to investigate the clinical manifestations and risk factors associated with PAL asthma.

Clinically, PAL asthma and Asthma‐COPD Overlap (ACO) syndrome are prone to confusion. ACO refers to a clinical state where both asthma and chronic obstructive pulmonary disease (COPD) coexist in the same patient. Notably, most scholars hold that ACO encompasses patients aged 40 years or older with a history of asthma or significant bronchodilator reversibility who present with PAL [6]. In contrast, other scholars emphasize the need to incorporate smoking history into the definition, as it plays a critical role in ACO patients [7]. Overall, a history of asthma and PAL are recognized as key definitional criteria for ACO [8]. Therefore, a substantial overlap exists between patients with ACO and those with PAL asthma. Specifically, some PAL asthma patients may fulfill the diagnostic criteria for ACO, but the two conditions are by no means entirely equivalent. PAL asthma is a severe phenotype of asthma.

Historically, asthma was viewed as predominantly affecting the large airways. However, a shift in understanding has identified the small airways as the primary site of airflow limitation [9]. Small airway dysfunction is present across all asthma severities and holds a prominent role in the pathophysiology of the disease [10]. Some investigators have categorized asthma based on small airway function and observed a close association between small airway function and asthma control, treatment outcomes, acute exacerbations, and patients' quality of life [11]. Kraft et al. [12] conducted a multifactorial analysis that underscored the significant correlation between MMEF25% and 75% (forced expiratory flow between 25% and 75% of forced vital capacity) and acute exacerbations as well as asthma control. MMEF25%–75% is recognized as a more precise indicator for assessing small airway function [13]. Additionally, research suggests that small airway dysfunction has superior predictive value for the clinical response to biological therapies in severe eosinophilic asthma compared to airflow obstruction [13]. Nevertheless, investigations into the interrelation between PAL asthma and small airway function are currently lacking.

Hence, our study aims to assess the clinical features of PAL asthma within this cohort and investigate the associated risk factors. Concurrently, we seek to explore the correlation between small airway function in asthma patients and the presence of PAL.

## 2 Methods

### 2.1 Study Design and Subjects

We conducted a retrospective analysis of clinical data from 1322 asthma patients diagnosed in the Department of Respiratory and Critical Care Medicine at shanghai ChangZheng hospital from January 1, 2018, to December 31, 2022. This analysis involved gathering information on clinical characteristics, pulmonary function tests, and blood routine test results of the asthma patients.

The clinical diagnosis was established by two attending physicians specializing in respiratory internal medicine, relying on patient medical history, clinical manifestations, laboratory assessments, pulmonary function tests, and bronchodilator responsiveness testing/bronchial provocation tests. The diagnosis primarily adhered to the criteria outlined in the Global Initiative for Asthma (GINA) guidelines, which combine clinical features with variable expiratory airflow limitation. PAL asthma was defined as asthma patients exhibiting a postbronchodilator forced expiratory volume in 1 s/forced vital capacity (FEV1/FVC) ratio below 0.7.

#### 2.1.1 Inclusion Criteria for Asthma Patients

Inclusion Criteria: (1) Patients aged 18–85 years. (2) Based on the 2022 GINA guidelines, patients diagnosed with asthma in our hospital from January 1, 2018, to December 31, 2022. (3) Complete pulmonary function testing and positive bronchial dilation test.

Exclusion criteria: (1) Patients with severe lung infections or pneumothorax during pulmonary function testing. (2) Pulmonary function test conducted during an acute exacerbation of asthma. (3) Incomplete examination of small airway function before and after bronchial dilation test or incomplete lung function test information. (4) Patients with malignant tumors, interstitial lung disease, or other autoimmune‐related diseases besides asthma. (5) Patients were diagnosed with COPD only.

The medical records to be compiled for the enrolled patients will encompass fundamental patient demographics (gender, age, height, weight, etc.), clinical presentations (wheezing, dyspnea, chest tightness, and cough), and laboratory investigations (white blood cell count, neutrophil count, eosinophil count, etc.).

### 2.2 Telephone Follow‐Up and Detection Methods

#### 2.2.1 Telephone Follow‐Up

All asthma patients underwent telephone follow‐up involving a questionnaire survey. The survey encompassed inquiries regarding smoking history, quantity of smoking, smoking cessation status, symptoms, duration since asthma diagnosis, current symptoms, medication utilization, history of lung cancer and other medical conditions, and the assessment of asthma control (well controlled, partly controlled, uncontrolled) and severity grading (mild, moderate, and severe) based on the patients' medical history and information gathered during the telephone follow‐up.

#### 2.2.2 Pulmonary Function Testing

All subjects underwent pulmonary function testing using a lung function testing instrument. The pulmonary function parameters included Forced Vital Capacity (FVC), Forced Expiratory Volume in 1 s (FEV1), FEV1/FVC ratio, Forced Expiratory Volume in 1 s percentage of predicted value (FEV1% predicted), Peak Expiratory Flow (PEF), Peak Expiratory Flow percentage of predicted value (PEF%), Residual Volume/Total Lung Capacity ratio (RV/TLC ratio), Total Respiratory Resistance (R tot), Total Specific Respiratory Resistance (SR tot), MMEF25%, MMEF50%, MMEF75%, and Maximum Mid‐Expiratory Flow between 25% and 75% of FVC (MMEF25%–75%).

#### 2.2.3 Bronchial Dilation Test

All participants underwent initial baseline lung function testing. Subsequently, upon successful completion of the baseline lung function assessment, they received an inhalation of 300 mcg of salbutamol and underwent a repeat lung function evaluation after 20 min. A positive bronchodilator response was defined as a postsalbutamol FEV1 increase exceeding 12% with an absolute increment greater than 200 mL. Participants refrained from using short‐acting bronchodilators for over 24 h, long‐acting bronchodilators for more than 48 h, short‐acting oral theophylline for over 12 h, and long‐acting oral theophylline for more than 48 h before the bronchodilator response test.

### 2.3 Statistical Methods

Statistical analysis was conducted using SPSS 26.0 software. Continuous variables were presented as mean ± standard deviation. The two‐sample independent *t*‐test was utilized for normally distributed continuous variables, whereas the nonparametric Mann–Whitney *U* test was applied for continuous variables that did not adhere to a normal distribution. Categorical variables were depicted as numbers (%) and assessed using the chi‐square test. Multifactorial logistic regression analysis and nomogram analysis were employed to construct a predictive model for identifying risk factors associated with PAL asthma. Multifactorial linear regression analysis was applied to identify important factors associated with ACT score. Statistical significance was set at *p* < 0.05.

## 3 Results

### 3.1 Clinical Features and Laboratory Examination Analysis

**FIGURE 1:**
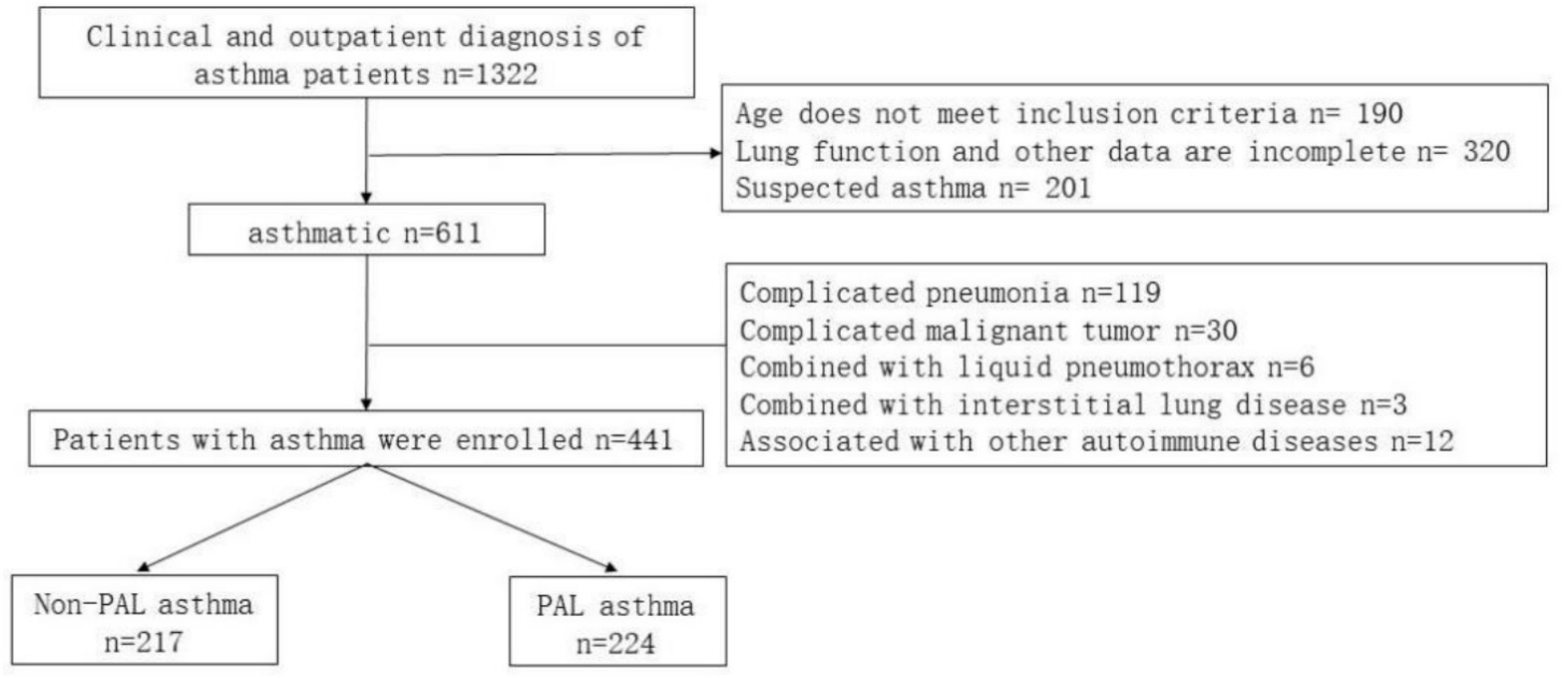
Screening process of enrolled asthmatic patients.

We conducted a retrospective analysis of clinical data from 1322 asthma patients, and a total of 521 patients were excluded due to missing pulmonary function data or suspected asthma diagnosis, along with 360 patients excluded due to age disparities and comorbidities like cancer, resulting in a final cohort of 441 asthma patients. By categorizing patients based on the presence of PAL, 217 individuals were classified as having nonpersistent airflow limitation (non‐PAL) asthma, while 224 patients were identified as having PAL asthma (Figure 1). Among them, 132 patients with non‐PAL asthma and 181 patients with PAL asthma had complete follow‐up data (Table 1).

**TABLE 1:** Comparisons of Basic clinical data and blood routine analysis between PAL and non‐PAL asthma groups.

	Non‐PAL asthma (*n* = 217)	PAL asthma (*n* = 224)	*p*
Male/female	122/95	182/42	< 0.01
Age	52.17 ± 17.14	59.87 ± 12.49	< 0.01
Height (cm)	165.53 ± 8.90	165.49 ± 7.61	0.76
Weight (Kg)	69.22 ± 12.95	68.55 ± 12.39	0.54
BMI (kg/m^2^)	25.22 ± 4.05	24.96 ± 3.73	0.54
**Follow‐up**	*n* = 132	*n* = 181	
Smoking history	81(61.4)	151(83.4)	< 0.01
Smoking index	350 ± 240	650 ± 298	< 0.01
Quit smoking	56(69.1)	47(26.0)	< 0.01
**Symptom**			
Wheezing	85(64.4)	158(87.3)	—
Shortness of breath	54(41.0)	132(72.9)	—
Chest distress	32(24.2)	120(66.3)	—
Cough	42(31.8)	92(50.8)	—
**Medical history**			
Allergic history	13(9.8)	20(11.05)	—
Onset of asthma (years)	8.9 ± 8.5	11.1 ± 10.2	0.06
ACT score	23.79 ± 2.53	19.86 ± 4.45	< 0.01
**Asthma control**			
Well Controlled	106(80.3)	82(45.3)	—
Partly controlled	15(11.4)	54(29.8)	—
Uncontrolled	11(8.3)	45(24.9)	—
**Asthma severity**			
Mild	91(68.9)	83(45.9)	—
Moderate	33(25.0)	58(32.0)	—
Severe	8(6.1)	40(22.1)	—
**Blood test**	*n* = 152	*n* = 191	
Leukocyte (×10^9^)	7.66 ± 3.24	7.55 ± 3.29	0.79
Neutrophil (×10^9^)	5.30 ± 3.46	5.19 ± 3.30	0.78
Eosinophil (×10^9^)	0.28 ± 0.76	0.26 ± 0.50	0.75

*Note:* The age matching was normal distribution and was analyzed by *t*‐test of two independent samples; the rest were not normal distribution and were analyzed by nonparametric Mann–Whitney *U* test. The categorical variables were analyzed by chi‐square test. Data are shown as the mean ± standard deviation or number (%).

Abbreviation: BMI, body mass index.

Patients with PAL asthma had a significantly higher mean age (59.87 years) than those with non‐PAL asthma (52.17 years), with a higher prevalence among male individuals (81.25% vs. 56.22%, *p* < 0.01). There was no statistically significant difference in height, weight, and BMI between the two cohorts. Individuals with PAL asthma demonstrated a higher frequency of smoking history (83.4% vs. 61.4%, *p* < 0.01) and smoking index (650 ± 298 vs. 350 ± 240, *p* < 0.01) in contrast to non‐PAL asthma patients, while the smoking cessation rate was higher in the non‐PAL asthma compared to the PAL asthma group (69.1% vs. 26.0%, *p* < 0.01). The predominant respiratory symptoms among asthma patients encompassed wheezing (64.4% vs. 87.3%), shortness of breath (41.0% vs. 72.9%), chest distress (24.2% vs. 66.3%), and cough (31.8% vs. 50.8%), observed in both non‐PAL and PAL asthma cohorts. However, PAL asthma individuals exhibited a heightened incidence of concurrent multiple symptoms compared to non‐PAL asthma patients, indicating a more pronounced symptomatology in PAL asthma (Table 1).

**FIGURE 2:**
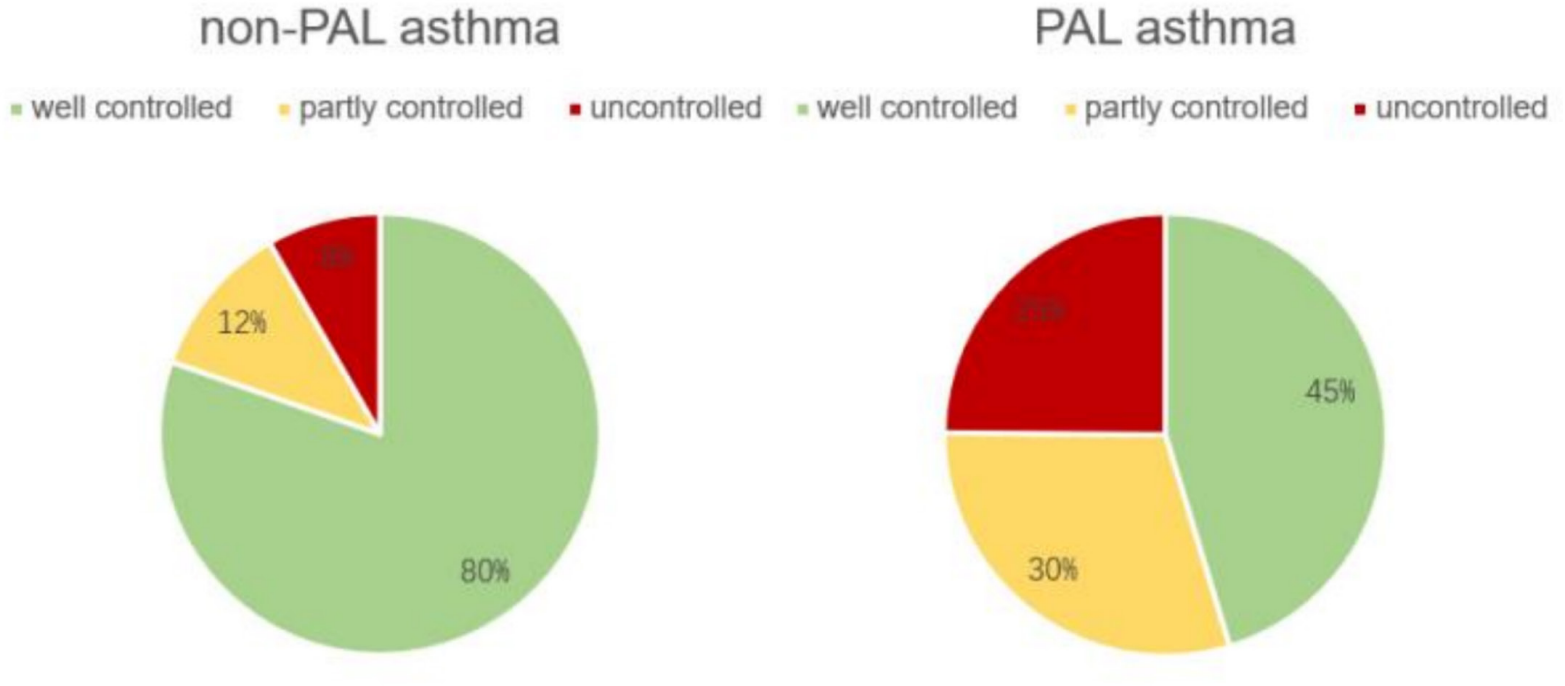
Severity classification of asthma patients. (A) Grading of severity of patients with PAL asthma; (B) severity grading of patients with non‐PAL asthma.

**FIGURE 3:**
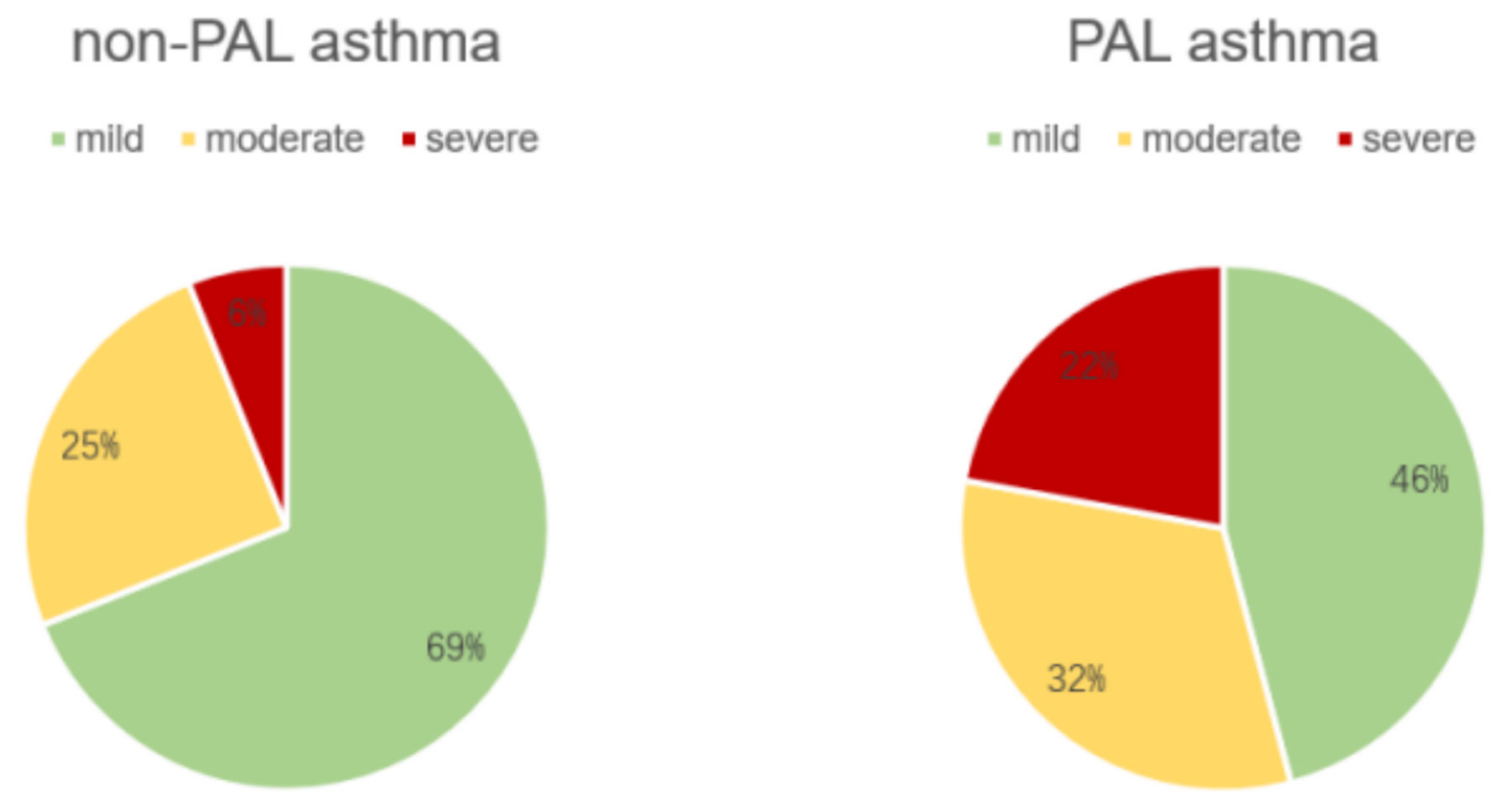
Asthma control of PAL and non‐PAL asthma patients. (A) Asthma control in patients with PAL asthma; (B) asthma control in patients with non‐PAL asthma.

In comparison to non‐PAL asthma individuals, there was no statistically significance in the duration of asthma (8.9 years vs. 11.1 years, *p* = 0.06) and a history of allergies (9.8% vs. 11.05%) in patients with PAL asthma. Notably, in the follow‐up Asthma Control Test (ACT) scores, non‐PAL asthma patients exhibited significantly higher scores than PAL asthma patients (23.79 vs. 19.86, *p* < 0.01), signifying a superior quality of life and asthma management in non‐PAL asthma (Table 1). As anticipated, non‐PAL asthma patients demonstrated better asthma control in comparison to PAL asthma patients. The prevalence of uncontrolled asthma was notably higher in PAL patients (24.9% vs. 8.3%) than in non‐PAL patients, while well controlled asthma was more prevalent in non‐PAL asthma individuals (Figure 2). In severity of asthma, the percentage of moderate (32.0% vs. 25.0%) and severe (22.1% vs. 6.1%) asthma cases were significantly higher in PAL patients than in non‐PAL asthma patients, with a lower occurrence of mild asthma (45.9% vs. 68.9%) (Figure 3). Blood tests, encompassing white blood cell counts, neutrophils, and eosinophils, revealed no statistically significant variances between the two groups (Table 1).

### 3.2 Analysis of Pulmonary Function and Bronchodilation Test

**FIGURE 4:**
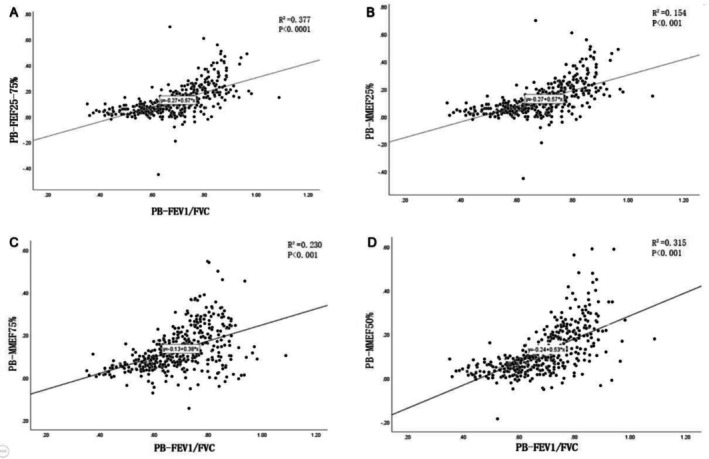
Scatter plot of FEV1/FVC and small airway function postbronchodilator in asthmatic patients. (A) PB‐MMEF25%–75% and PB‐FEV1/FVC scatter plots indicate that PB‐MMEF25%–75% is positively correlated with PB‐FEV1/FVC (*p* < 0.0001); (B) PB‐MMEF25% and PB‐FEV1/FVC scatter plots indicate that PB‐MMEF25% is positively correlated with PB‐FEV1/FVC (*p* < 0.001); (C) PB‐MMEF75% and PB‐FEV1/FVC scatter plot indicated that PB‐MMEF75% was positively correlated with PB‐FEV1/FVC (*p* < 0.001); (D) PB‐MMEF50% and PB‐FEV1/FVC scatter plot indicates that PB‐MMEF50% is positively correlated with PB‐FEV1/FVC (*p* < 0.001).

Patients with PAL asthma displayed significant obstructive ventilation impairment both pre‐ and postbronchodilator inhalation, characterized by more pronounced small airway dysfunction, compromised lung diffusion function, and increased residual volume in comparison to non‐PAL asthma. Patients with PAL asthma exhibited more severe obstructive ventilation impairment than non‐PAL asthma patients (FEV1/FVC 0.55 vs. 0.72, *p* < 0.01), with no substantial alleviation of obstructive ventilation impairment postbronchodilator inhalation (PostBronchodilator FEV1/FVC = 0.58 ± 0.07) (Table 2). Neither cohort demonstrated restrictive ventilation impairment but displayed a notable rise in residual volume. PAL asthma patients had notably elevated residual volume relative to non‐PAL asthma patients (RV/TLC 0.64 vs. 0.6, *p* < 0.01). Both groups exhibited significant impairment in lung diffusion function, with PAL asthma patients manifesting more severe impairment than non‐PAL asthma patients (DLCO% 0.59 vs. 0.71, *p* < 0.01) (Table 2). Significant small airway dysfunction was evident in both asthma groups, patients with PAL asthma demonstrating more severe dysfunction compared to non‐PAL asthma patients (MMEF25% 0.25 vs. 0.45, *p* < 0.01; MMEF50% 0.20 vs. 0.47, *p* < 0.01; MMEF75% 0.24 vs. 0.61, *p* < 0.01; MMEF25–75% 0.21 vs. 0.44, *p* < 0.01) (Table 2). Scatter diagrams revealed a positive correlation between FEV1/FVC and small airway function in asthma patients' postbronchodilator inhalation (Figure 4).

**TABLE 2:** Comparisons of lung function between patients with PAL and non‐PAL asthma groups.

	Non‐PAL asthma (*n* = 217)	PAL asthma (*n* = 224)	*p*
FEV1%pred	0.71 ± 0.16	0.49 ± 0.14	< 0.01
FVC%	0.86 ± 0.18	0.80 ± 0.15	< 0.01
FEV1/FVC	0.72 ± 0.06	0.55 ± 0.08	< 0.01
MMEF25%	0.45 ± 0.21	0.25 ± 0.13	< 0.01
MMEF50%	0.47 ± 0.19	0.2 ± 0.08	< 0.01
MMEF75%	0.61 ± 0.4	0.24 ± 0.11	< 0.01
MMEF25%–75%	0.44 ± 0.18	0.21 ± 0.09	< 0.01
PEF%	0.73 ± 0.19	0.49 ± 0.19	< 0.01
RV/TLC	0.53 ± 0.12	0.64 ± 0.08	< 0.01
DLCO%	0.71 ± 0.22	0.59 ± 0.23	< 0.01
R tot	0.60 ± 0.33	0.82 ± 0.38	< 0.01
SR tot	2.67 ± 1.37	5.05 ± 2.87	< 0.01
PB‐FEV1%pred	0.84 ± 0.18	0.6 ± 0.15	< 0.01
PB‐FEV1/FVC	0.78 ± 0.06	0.58 ± 0.07	< 0.01
PB‐MMEF25%	0.63 ± 0.29	0.32 ± 0.14	< 0.01
PB‐MMEF50%	0.64 ± 0.22	0.26 ± 0.1	< 0.01
PB‐MMEF75%	0.63 ± 0.24	0.28 ± 0.15	< 0.01
PB‐MMEF25%–75%	0.76 ± 0.23	0.33 ± 0.13	< 0.01
PB‐PEF%	0.82 ± 0.18	0.56 ± 0.15	< 0.01

*Note:* Data are shown as the mean ± standard deviation.

Notably, we conducted a comparison of differences in obstructive ventilation impairment and small airway function parameters, such as FEV1/FVC, PEF%, MMEF75%, MMEF25%–75%, before and after bronchodilator inhalation between patients diagnosed with PAL asthma and non‐PAL asthma. PAL asthma patients exhibited a slightly reduction in the rate of FEV1 improvement compared to non‐PAL asthma individuals. Importantly, there was a significant decrease in the improvement of small airway‐related indicators following bronchodilator inhalation, including the PB‐MMEF25%–75% remission rate (0.184 ± 0.118 vs. 0.071 ± 0.125, *p* < 0.01), PB‐MMEF75% remission rate (0.175 ± 0.110 vs. 0.085 ± 0.056, *p* < 0.01), PB‐MMEF50% remission rate (0.174 ± 0.132 vs. 0.062 ± 0.049, *p* < 0.01), PB‐MMEF25% remission rate (0.180 ± 0.243 vs. 0.066 ± 0.110, *p* < 0.01) (Table 3).

**TABLE 3:** Comparisons of pulmonary function postbronchodilation between patients with PAL and non‐PAL asthma.

	Non‐PAL asthma (n = 217)	PAL asthma (n = 224)	*p*
PB‐FEV1‐RR	0.129 ± 0.051	0.111 ± 0.046	< 0.01
PB‐FEV1‐RV	0.375 ± 0.163	0.313 ± 0.122	< 0.01
PB‐FEV1/FVC‐RR	0.059 ± 0.051	0.031 ± 0.044	< 0.01
PB‐PEF‐RR	0.087 ± 0.067	0.073 ± 0.136	0.18
PB‐PEF‐RV	0.701 ± 0.57	0.637 ± 0.874	0.37
PB‐MMEF25%–75%‐RR	0.184 ± 0.118	0.071 ± 0.125	< 0.01
PB‐MMEF25%–75%‐RA	0.628 ± 0.431	0.218 ± 0.389	< 0.01
PB‐MMEF75%‐RR	0.175 ± 0.110	0.085 ± 0.056	< 0.01
PB‐MMEF75%‐RA	1.214 ± 0.833	0.604 ± 0.411	< 0.01
PB‐MMEF50% ‐RR	0.174 ± 0.132	0.062 ± 0.049	< 0.01
PB‐MMEF50%‐RA	0.728 ± 0.554	0.249 ± 0.192	< 0.01
PB‐MMEF25%‐RR	0.180 ± 0.243	0.066 ± 0.110	< 0.01
PB‐MMEF25%‐RA	0.269 ± 0.351	0.073 ± 0.133	< 0.01

*Note:* ‐RR = remission rate (e.g., PB‐FEV1‐RR = PB‐FEV1%pred‐FEV1%pred); −RA = Remission value (e.g., PB‐FEV1‐RA = PB‐FEV1‐FEV1); data are shown as the mean ± standard deviation.

### 3.3 Multivariate Logistic Regression Analysis of PAL Asthma

We conducted a multivariate logistic regression analysis of the basic patient data among asthmatic patients, comparing the characteristics and laboratory results of PAL asthma patients (*n* = 181) and non‐PAL asthma patients (*n* = 132). We found statistical significance in gender, age, smoking index, and smoking status between the two groups. By performing multifactorial logistic regression analysis on patient gender, age, smoking index, smoking status, and ACT scores, setting an inclusion criterion of 0.05 and an exclusion criterion of 0.1, we identified gender, smoking index, and ACT scores as independent risk factors for PAL asthma with statistical significance (*p* < 0.05) (Table 4). The predictive accuracy of this study model was 89.1%, indicating acceptable model fit (Figure 6).

**TABLE 4:** Results of multivariate logistic regression analysis.

	B	Standard error	Wald	*p*	OR	95%CI
Gender	0.812	0.368	4.871	0.027	2.253	1.095–4.634
Smoking index	0.003	0.001	28.755	< 0.001	1.003	1.002–1.004
ACT	−0.352	0.053	43.947	< 0.001	0.703	0.634–0.780

Abbreviation: ACT, asthma control test.

**FIGURE 5:**
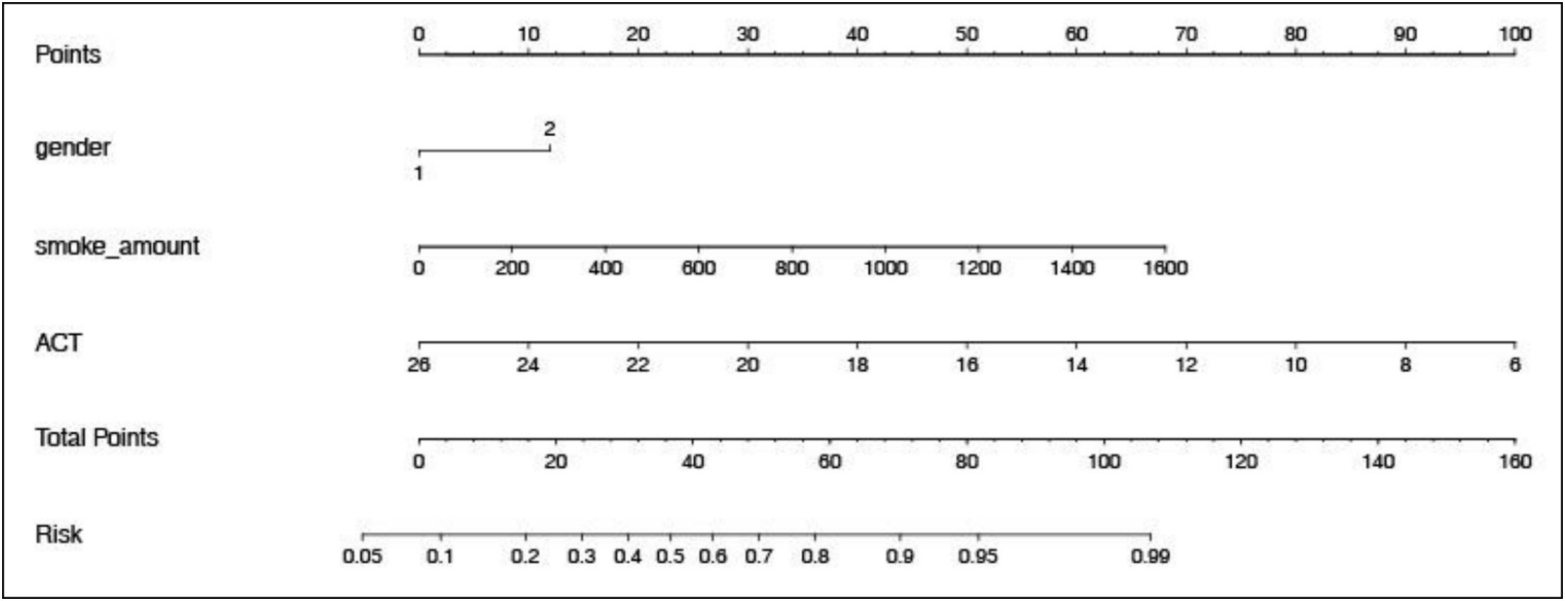
Nomogram of the logistic regression model predicting PAL asthma. For example, a male asthma patient whose smoking index was 800 and had an ACT score of 18 would receive a score of 11 + 31 + 40 = 82 points, indicating a 96% risk of developing PAL asthma.

**FIGURE 6:**
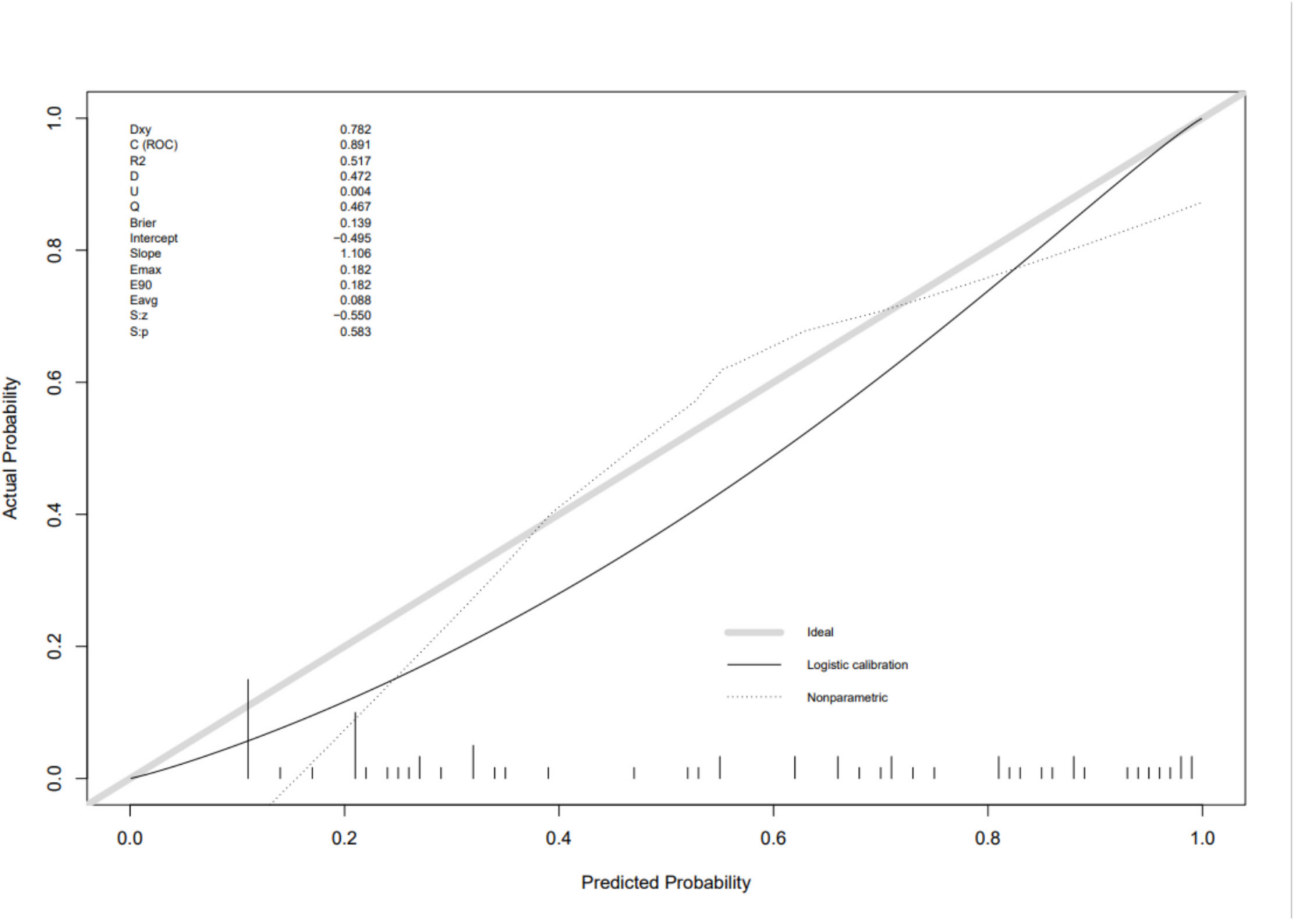
Calibration diagram of logistic regression model.

Based on the results of the multivariate logistic analysis, we constructed a line graph to make the developed predictive model for PAL asthma more concrete and intuitive for clinical application (Figure 5). Figure 6 shows a C‐index of 0.891, indicating good predictive capability for the established model.

Calibration serves as an assessment of the precision of a disease risk model in forecasting occurrence probabilities, showcasing the alignment between the model‐predicted disease risk and the actual disease incidence. Figure 6 illustrates the calibration plot of the logistic regression model for predicting PAL asthma. The plot reveals favorable consistency in predicting both low and high‐risk disease probabilities, signifying a strong concordance between the estimated risk and the observed prevalence of PAL asthma.

The Hosmer and Lemeshow Test resulted in a *p*‐value of 0.583, indicating a good fit of the predictive formula for PAL asthma. This test assesses the significance of differences between the predicted values derived from the model and the actual observed data, with *p* > 0.05 indicating a high level of adequacy in model fitting (Figure 6).

### 3.4 Multivariate Linear Regression Analysis of ACT Score in Asthma

**FIGURE 7:**
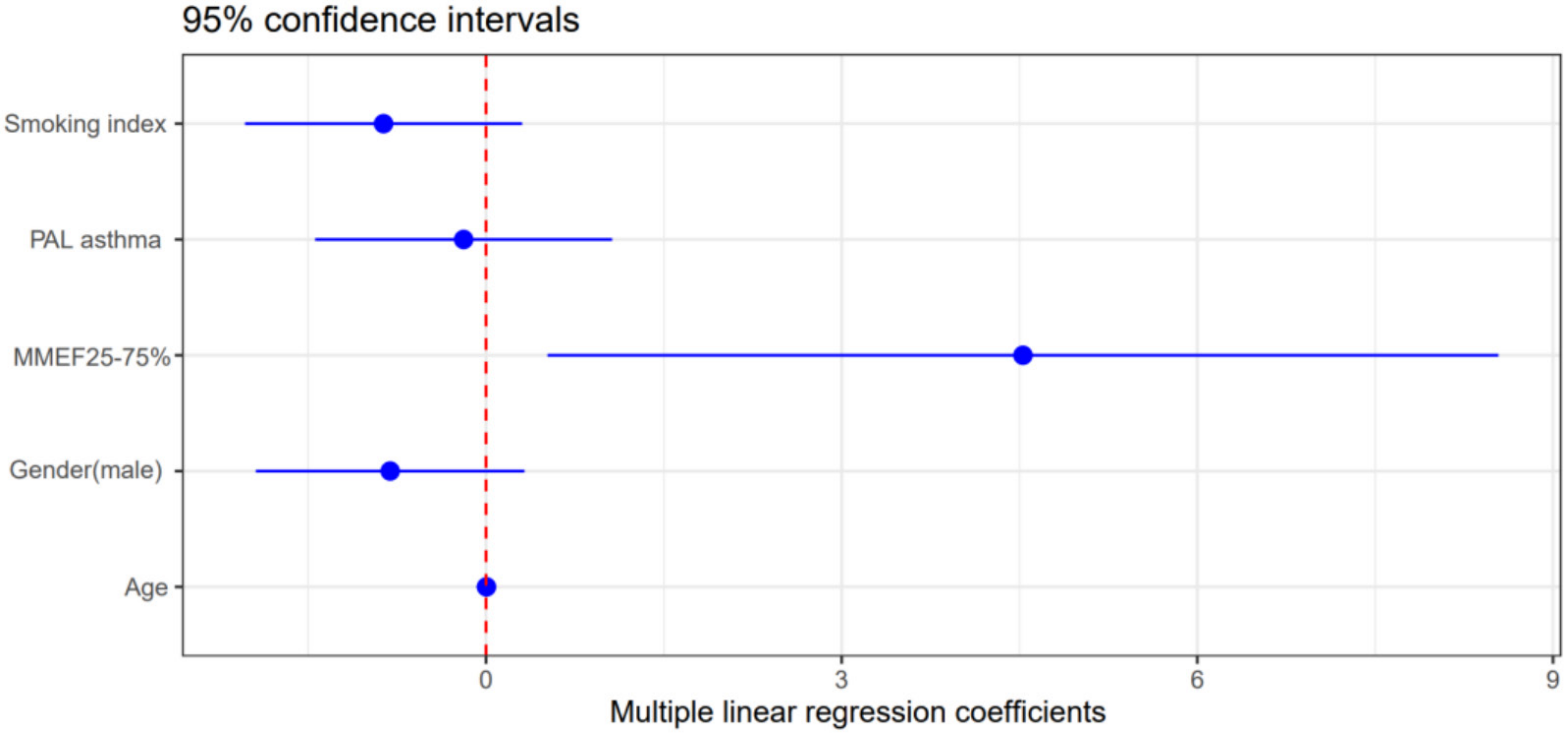
Multiple linear regression coefficients and 95% confidence intervals. Set the ACT score as the dependent variable, and MMEF25%–75% was significantly positively correlated with the outcome.

The PAL asthma, smoking index, and male gender were negatively correlated with the ACT score. However, in the *p*‐values of the multiple linear regression results, they were all lower than 0.05, indicating a relatively low correlation (Table 5). In contrast, MMEF25%–75% was significantly positively correlated with the ACT score. This correlation was significantly higher than that of factors such as age, gender, PAL asthma, and smoking (Figure 7). This indicates that small airway function is an important independent factor influencing the quality of life of asthma patients.

**TABLE 5:** Results of multiple linear regression analysis.

Factors	Regression coefficient	*p*
Constant	21.300	0.000
Smoking index	−0.865	0.147
MMEF25%–75%	4.530	0.027
PAL asthma	−0.189	0.767
Age	0.004	0.847
Gender (male)	−0.809	0.161

## 4 Discussion

Our investigation revealed a higher prevalence of PAL asthma in elderly male patients compared to non‐PAL asthma. The proportion of male patients in PAL asthma was notably elevated at 81.25%, contrasted with 56.22% in non‐PAL asthma. Furthermore, advancing age emerged as a potential high‐risk factor for PAL asthma, aligning with observations by Kole et al. [3]. Previous studies have highlighted that late‐onset adult asthma patients exhibit higher asthma symptom burden and mortality rates relative to younger counterparts [14]. Correspondingly, Zhang et al. [15] documented that elderly asthma patients experience poorer asthma control, often falling into the moderate to severe classification, reflecting lower rates of diagnosis and treatment among elderly late‐onset adult asthma individuals [11]. Although our study indicated a lengthier duration of asthma in PAL asthma patients compared to non‐PAL asthma, the difference was not statistically significant. Given that the asthma duration data were obtained partially via telephone follow‐up, potential bias may exist in this information. Therefore, further large‐scale prospective clinical investigations are warranted to validate the association between late‐onset adult asthma and PAL asthma.

Our findings suggest that smoking could be a significant factor in the development of PAL asthma. The prevalence of smoking among PAL asthma patients was notably higher compared to non‐PAL asthma patients (83.4% vs. 61.4%, *p* < 0.01), with PAL asthma patients also having a higher smoking index. Previous research has indicated that both active and passive exposure to smoking in individuals, including children and adults, can elevate the risk of asthma development [16]. Additionally, smokers with asthma face an increased likelihood of developing COPD compared to nonsmokers [17]. Therefore, cessation or reduction of smoking habits may serve as an effective strategy in mitigating the risk of PAL asthma. Notably, given the higher prevalence of smoking in males, a study in Kuwait revealed a substantially higher smoking history among males (49.9%) than females (4.4%) [18], potentially contributing to the elevated incidence of PAL asthma in males. Ceasing smoking habits may offer a valuable approach in preventing asthma patients from experiencing PAL. In our investigation, the rate of smoking cessation was significantly higher in non‐PAL asthma patients (69.1%) compared to PAL asthma patients (26.0%). Research conducted by Tønnesen et al. has demonstrated that smoking cessation can enhance the quality of life for asthma patients and alleviate asthma symptoms [19]. Similarly, Chaudhuri et al. [20] reported improved lung function and reduced sputum eosinophil counts in asthma patients who quit smoking 6‐week postcessation. Consequently, reducing or quitting smoking is a crucial complementary intervention for asthma patients [21].

Additionally, our investigation demonstrated that PAL asthma patients not only displayed severe obstructive ventilation impairment but also exhibited elevated residual volume, aligning with the findings reported by Kole et al. [3]. We identified noteworthy deficits in lung diffusion function among PAL asthma patients, consistent with the observations made by Kraemer et al. [22], who noted impaired lung diffusion function in asthma patients with COPD. Particularly striking was the more pronounced difference in small airway dysfunction between PAL and non‐PAL asthma patients compared to other pulmonary function parameters. Small airways typically refer to air passages with an inner diameter less than 2 mm, commonly encompassing the 8th generation bronchi and lower branches within the bronchial tree [23]. Xiao et al. [24] documented that over 40% of Chinese adults exhibit small airway dysfunction, a condition prevalent in both asthma and COPD. Within our study cohort, small airway dysfunction was prevalent among asthma patients, with PAL asthma patients demonstrating more severe dysfunction in the small airways. A scatter plot analysis revealed a significant positive correlation between MMEF25% and 75% and the ratio of FEV1/FVC following bronchodilator administration. In asthma patients, poorly controlled inflammation in the small airways is linked to accelerated airway remodeling and a decline in lung function, underscoring the diagnostic significance of the small airways in asthma management [25]. Recent research has highlighted chronic inflammation and functional impairment in the small airways as pivotal risk factors for asthma severity, disease control, acute exacerbations, and age‐related decline in lung function [26]. In another prospective study, it was also found that small airway function assessed by MMEF25%–75% and impulse oscillometry (IOS) was significantly associated with asthma control and disease exacerbations. Notably, MMEF25%–75% and IOS exhibited similar assessment efficacy for small airway function in their research [12].

Abnormalities in small airway function often occur early in the development of asthma [27]. Consistent with this, our study revealed a significant correlation between small airway function and PAL asthma. However, limited by the retrospective study design, we were unable to conduct longitudinal observations to determine whether small airway function can predict the development of PAL asthma. Nevertheless, our findings provide a potential direction for the prediction of PAL asthma.

Our study revealed a notable difference in the rate of improvement in small airway function following bronchodilator inhalation between PAL asthma patients and non‐PAL asthma patients, with PAL asthma patients showing a significantly lower rate of improvement. In another study published in recent years, it was found that obese female asthmatic patients exhibited stronger small airway responsiveness during bronchial provocation tests, which underscores the crucial value of small airway responsiveness in the treatment and assessment of asthma [28]. This disparity implies that PAL asthma patients may encounter greater treatment challenges, suggesting that therapeutic interventions targeting small airway dysfunction could be particularly beneficial for this patient population. To date, there is a paucity of research addressing this specific clinical observation.

Papi et al. [4] reported that triple inhaled therapy involving fluticasone furoate/vilanterol/umeclidinium was more effective in normalizing airflow and reducing the risk of acute exacerbations in PAL asthma patients compared to dual inhaled therapy with fluticasone furoate/vilanterol. Similarly, Van Zyl‐Smit et al. [29] also found triple inhaled therapy to be more efficacious for PAL asthma patients than dual inhaled therapy, yet only a subset of PAL asthma patients achieved optimal asthma control. Studies have indicated that utilizing smaller aerosol particles for peripheral airway medication delivery yields superior outcomes compared to larger particle medications, resulting in decreased daily doses of inhaled corticosteroids and enhanced asthma control, thereby augmenting quality of life [30]. In light of our study findings, triple therapy incorporating small aerosol particles could represent an effective treatment approach for PAL asthma. Previous studies have indicated that particle size and distribution are crucial when developing inhaled therapies for asthma; however, the development and application of drugs targeting the small airways in asthma remain controversial [31]. Our study may suggest that small airway responsiveness varies among different asthma phenotypes, and thus, treatments capable of reaching the small airways are more critical for PAL asthma.

Moreover, the diminished rate of small airway improvement postbronchodilator inhalation in PAL asthma patients warrants heightened attention, as it may serve as a valuable indicator for tailored medication management in individuals with PAL asthma.

In summary, we developed a predictive model utilizing general patient data to forecast the probability of asthma patients being diagnosed with PAL asthma. This model demonstrates a high level of accuracy in predicting the likelihood of PAL asthma among asthma patients, offering valuable insights for clinical management. Notably, smoking emerged as a key contributing factor for PAL asthma patients.

Limitations of this study: (1) Being a retrospective investigation, patient medical records was reliant on the medical records system retrieval and telephone follow‐up, potentially introducing bias into the data collected. (2) Conducting the study at a single center may lead to selection bias in patient enrollment. (3) In this study, MMEF25%–75% and body plethysmography were employed to assess small airway function. Techniques such as IOS enable more precise assessment of small airway function, while chest computed tomography (CT) has also garnered increasing attention for small airway evaluation. Thus, more accurate methods for small airway assessment may be required to verify the findings of this study in future researches. To enhance the credibility of information and assess patient medication compliance, further prospective studies are warranted to investigate acute exacerbations and medication‐related issues in PAL asthma.

## 5 Conclusion

PAL asthma patients demonstrate inadequate asthma control, decreased quality of life, and a higher likelihood of being categorized as moderate to severe asthmatics. Notably, PAL asthma can also manifest in individuals with mild asthma. PAL asthma is more prevalent among elderly male patients. Utilizing multifactorial logistic regression analysis, a predictive model was constructed for PAL asthma, indicating that gender, smoking history, smoking index, and ACT scores serve as independent risk factors for PAL asthma. Smoking cessation may represent an effective strategy for PAL asthma patients to ameliorate symptoms and enhance treatment effectiveness. Furthermore, PAL asthma patients display pronounced small airway dysfunction, with minimal improvement in small airway function postbronchodilator inhalation, underscoring the potential value of interventions targeting small airway dysfunction in the management of PAL asthma.

## Author Contributions

Zhongzhao Wang and Yang Luo contributed to manuscript writing. Xintong Du and Yang Luo contributed to data collection; Zhongzhao Wang and Heng Gong contributed to statistical analysis. Hao Tang contributed to the design of the experiment and reviewed the study.

## Funding

This study is sponsored by the “Shuguang Program” supported by the Shanghai Education Development Foundation; Shanghai Municipal Education Commission (20SG38), Shanghai Municipal Science and Technology Committee of Shanghai Outstanding Academic Leaders Plan (20XD1423300); General Program of National Nature Science Foundation of China (No. 82070036); The “Deep Blue” Project ‐“Qianghai” Innovation Team of “Deep Blue” Project (Naval Medical University)—SL44.

## Ethics Statement

All procedures involving human participants were conducted in accordance with the Declaration of Helsinki and approved by the Ethical Committee of Changzheng Hospital, Navy Medical University, approval number (2024SL055). Informed consent was waived by the Ethical Committee due to the retrospective analysis of clinical data. The study involves human subjects and includes interaction with human subjects (obtaining surveys).

## Conflicts of Interest

The authors declare no conflicts of interest.

## Data Availability

Data that supports the findings of this study are available from the corresponding author.
